# Effect of miR-145 on intracardiac thrombosis in patients with rheumatic heart disease complicated with atrial fibrillation: A retrospective analysis

**DOI:** 10.1097/MD.0000000000033385

**Published:** 2022-04-07

**Authors:** Xiong Zhang, Kai Deng, Xu-Ai Ren, Xiao-Ming Su

**Affiliations:** a Department of Laboratory, First People’s Hospital of Xianyang, Shaanxi, China; b Department of Laboratory, First Hospital Affiliated to Xi’an Jiaotong University, Shaanxi, China; c Department of Cardiology, First People’s Hospital of Xianyang, Shaanxi, China.

**Keywords:** atrial fibrillation, miR-145, rheumatic heart disease, thrombosis

## Abstract

Patients with rheumatic heart disease (RHD) and atrial fibrillation (AF) often have a risk of intracardiac thrombosis. Exfoliated thrombus is easy to cause embolic diseases. This study revealed the risk of intracardiac thrombosis in patients with RHD with AF by exploring the expression of plasma microRNA miR-145. The expression of plasma miR-145 in 58 patients with RHD complicated with AF was detected by real-time quantitative polymerase chain reaction [28 cases in thrombus (TH) group and 30 cases in non-thrombus (NTH) group]. At the same time, a healthy control group (33 cases) was established. The correlation between miR-145 and thrombosis in RHD was analyzed. The expression of plasma miR-145 in TH group and NTH group decreased significantly, especially in TH group (*P* < .01). In TH group and NTH group, the expression of miR-145 was negatively correlated with D-Dimer level, Factor XI concentration and tissue factor level as well as left atrial diameter (all *P* < .01, respectively). The receiver operating curve analysis showed that the expression of miR-145 had diagnostic significance for RHD and its intracardiac thrombosis. In this study, we suggest that the change of plasma miR-145 expression in patients with RHD is related to coagulation activity and fibrinolysis, which can predict the risk of intracardiac thrombosis.

## 1. Introduction

Rheumatic heart disease (RHD) is an autoimmune disease caused by Streptococcus pyogenes infection. It is the main sequelae of acute rheumatic fever, and clinical data show that it can be prevented.^[1]^ Studies have shown that heart valve injury is caused by long-term recurrent rheumatic valvulitis. When atrial fibrillation (AF) occurs at the same time, thrombosis and stroke events increase significantly,^[2]^ especially left atrial thrombus shedding is easy to cause embolic diseases.^[3]^ microRNAs (miRNAs) are some small noncoding RNAs, which regulate gene expression at the post transcriptional level either by inhibiting messenger RNA (mRNA) translation or by promoting mRNA degradation.^[4]^ It is reported that several miRNAs, including miR-155, miR-126, miR-21, and miR-145, can regulate pathogenic signals in the development and progression of vascular inflammation, neointimal lesion formation, atherosclerosis and coronary artery disease.^[5–7]^ At present, the role of miRNAs in RHD has become a hot spot. Molecular technology can detect RHD early and treat it early. However, the molecular mechanism of miRNA affecting RHD cardiomyocyte apoptosis and remodeling has not been fully clarified.^[8]^ In this study, we used real-time quantitative polymerase chain reaction (qRT-PCR) to explore the possible role of miR-145 in intracardiac thrombosis in patients with RHD complicated with AF. Importantly, our results suggest that a decrease in miR-145 expression is associated with an increased risk of intracardiac thrombosis.

## 2. Materials and methods

### 2.1. Subjects

This study was approved by the medical ethics committee of First Affiliated Hospital of Xi’an Jiaotong University and First people’s hospital of Xianyang city. All participants obtained informed consent. The study was conducted in accordance with the Helsinki declaration.

**Figure 1.**
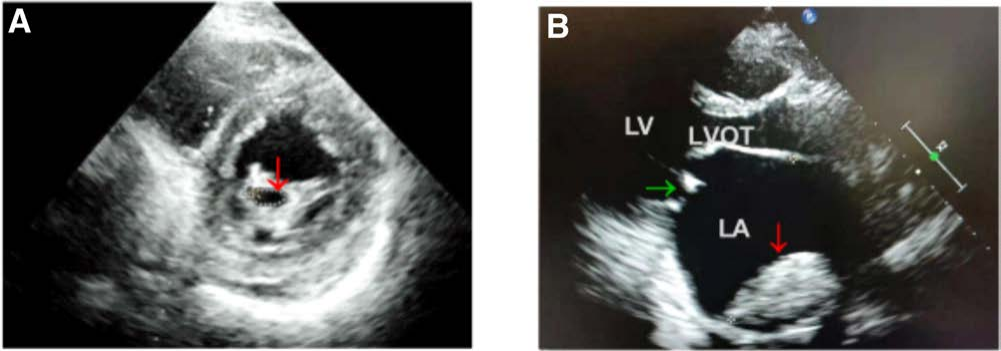
Echocardiographic confirmation of RHD patients. RHD = rheumatic heart disease.

The clinical data of 58 patients with RHD hospitalized in the Department of Cardiology in our hospital from July 2017 to July 2020 were collected and analyzed retrospectively. There were 48 cases of mitral valve disease and 10 cases of combined mitral and aortic valve disease, all of which were complicated with AF. The cardiac function grade (New York Heart Association) of all patients was grade II to III. All patients were examined by echocardiography. According to whether there was thrombosis in the heart, they were divided into thrombus (TH) group and non-thrombus (NTH) group. The thrombus group included 28 patients, including 22 cases of left atrial appendage thrombus and 6 cases of left ventricular thrombus, and 30 cases in the NTH group. Exclusion criteria: Patients with coronary heart disease and congenital heart disease; Hyperthyroid heart disease and core pulmonale; Left ventricular ejection fraction below 40%; Those with hypertension and diabetes. Patients with a history of heart surgery, tumor, cardiomyopathy, nephritis, renal failure, and gout. RHD was diagnosed through the clinical manifestations and echocardiographic results of patients on the basis of the standards of the World Heart Federation.^[9]^ The diagnosis of AF was confirmed by ECG and Holter. Figure 1A showed a mitral stenosis in the patient with RHD, and Figure 1B showed a left atrial thrombosis and a significantly enlarged left atrium in the patient with RHD. Thirty-three healthy persons from the physical examination center in the same period were selected as the control group. Thrombosis and family history were not presented. No medication was used for two weeks.

### 2.2. Methods

Clinical data (gender, age and blood pressure) were collected for all participants. The left atrial diameter (LAD) and heart valve lesions in patients with RHD were analyzed by GE ultrasonic diagnostic instrument. The expression of mirna-145 was detected by qRT-PCR. D-Dimer (D-D) level, Factor XI Factor XI concentration (FXI: C) and tissue factor (TF) level as well as LAD for RHD patients were analyzed.

### 2.3. Specimen collection

After 12 hours of fasting for All participants, 10 mL of venous blood was collected and divided into two tubes (4 mL and 6 mL). One tube of blood was not anticoagulated, and another 6 mL of blood was anticoagulated with sodium citrate, the ratio of blood to anticoagulant was 9:1. All blood was centrifuged at a speed of 3000 rpm for 15 minutes and then separated into serum or plasma. D-D level determination was completed within 2 hours, and another 2 mL of plasma was taken and stored at −80 °C for standby.

### 2.4. Research methods

The serum levels of interleukin-6 (IL-6) and TF were detected by enzyme-linked immunosorbent assay (ADL co., USA). and the Infinite 20 Pro multifunctional enzyme labeling instrument was purchased from TECAN company in Switzerland. A sysmex7000 automatic hemagglutination analyzer (Sysmex, Japan) was used to detect the levels of D-D and Factor XI concentration. The reagent adopts the reagent kit of Siemens company in Germany.

### 2.5. MiR-145 expression detection

Plasma total RNA was extracted using Trizol Kit (Invitrogen, CA) according to the manufacturer’s instructions. The absorbance of the extract at 260 and 280 nm was determined by Ultraviolet spectrophotometry. The ratio between 1.7 and 2.1 indicated that the sample had high purity and could be used for further research. RNA was reverse transcribed into cDNA using a reverse transcription kit (Yiqiao Shenzhou Technology Co., Ltd, Beijing, China). qRT-PCR used an ABI 7500 quantitative PCR system (Thermo Fisher Scientific, Waltham, MA). β-actin was used as an internal reference. The primer sequence of miR-145 was: Forward: 5’-GCCTCCCTGAGACCCTTTA-3’, Reverse: 5’- GTGTCGTGGAGTCGCA-3’. The primer sequence of β-actin was Forward: 5’-AGGCACTGGGCTTCATCTGAC-3’, Reverse: 5’- GCCTTCCATCCTTGCTTAG-3’. PCR reaction conditions: Pre denaturation at 95 °C for 1 minute; denaturation at 95 °C for 5 seconds, anneal at 60 °C for 10 seconds, extension at 72 °C for 15 seconds (collect fluorescence), and repeating 40 cycles. The amplified products were electrophoresed. The absorbance of miR-145 and β-actin was obtained by Ultraviolet spectrophotometry. The absorbance of β-actin as the reference, calculated the change value of absorbance of miR-145 (ΔCT), and the relative expression of miR-145 was obtained by 2^-ΔCT^.

### 2.6. Statistical analysis

The data were analyzed by SPSS 20.0 statistical software (IBM Corp., Chicago, IL), and the data were expressed as mean ± standard deviation. The measurement data among the groups were compared by 1-way ANOVA test with a post hoc Tukey’s test, and the count data were compared by χ ^2^ test. Pearson method was used for a correlation analysis. Logistic regression was used to analyze the risk factors of intracardiac thrombosis in patients with RHD and expressed by odds ratio (OR) and 95% confidence interval (CI). receiver operating curve (ROC) was established to evaluate the clinical significance of plasma miR-145 expression in the diagnosis of thrombosis for RHD patients. The difference was statistically significant (*P* < .05).

## 3. Results

### 3.1. Comparison of baseline data between the groups

**Figure 2.**
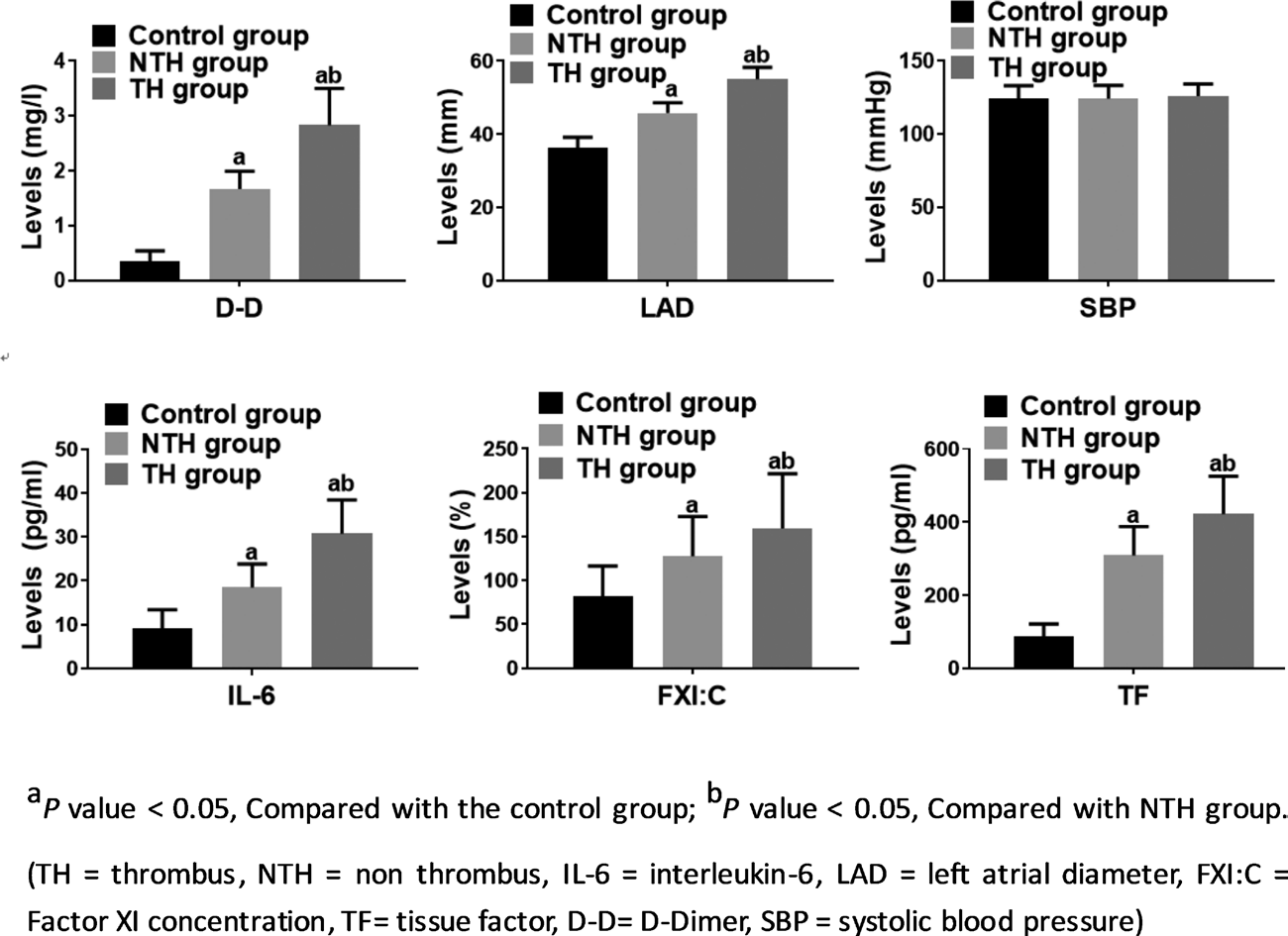
Confirmation of laboratory and ultrasonic examination.

Our results indicated that LAD in the control group, NTH group and TH group showed a gradual increasing trend (all *P* < .05, Table 1 and Figure 2). No significant differences were found in age, sex, and systolic blood pressure among the groups (all *P >* .05, Table 1).

**Table 1:** Comparison of general data among the groups.

Items	Control group (N = 33)	NTH group (N = 30)	TH group (N = 28)	*P* value
Male (cases)	12	12*	14*^,^†	>.05
Age (*x̅*±s, years)	36.29 ± 13.53	35.32 ± 14.12*	35.66 ± 13.97*^,^†	>.05
LAD (*x̅*±s, mm)	36.32 ± 2.9	45.93 ± 2.71‡	55.37 ± 3.23‡^,^§	<.05
SBP (*x̅*±s, mm Hg)	124.5 ± 8.7	124.9 ± 8.9*	126.1 ± 8.3*^,^†	>.05

LAD = left atrial diameter, NTH = non thrombus, SBP = systolic blood pressure, TH = thrombus, x̄±s = mean ± standard deviation.

* *P* value > .05, Compared with the control group.

† *P* value > .05, Compared with NTH group.

‡ *P* value < .05, Compared with the control group.

§ *P* value > .05 Compared with NTH group.

### 3.2. Laboratory analysis

**Figure 3.**
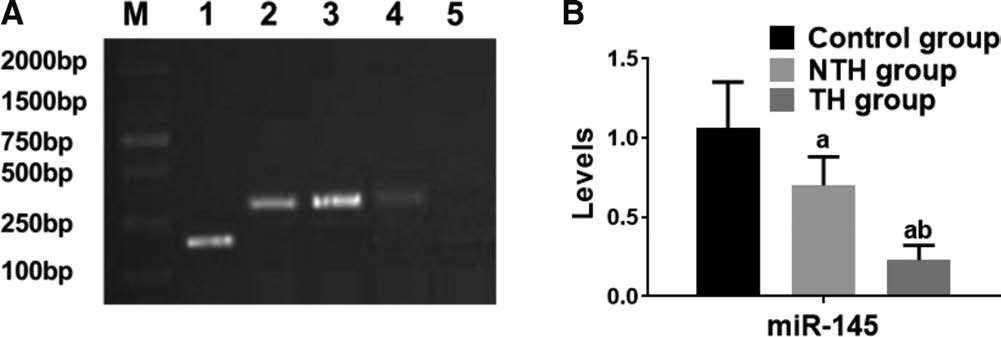
Determination of miR-145 expression in RHD patients. RHD = rheumatic heart disease.

Our results showed that the levels of D-D, TF, IL-6, and FXI: C increased gradually (all *P >* .05, Table 2, Fig. 2) whereas miR-145 expression decreased gradually (all *P* < .05, Table 2, Fig. 3) in the control group, NTH group and TH group.

**Table 2:** Laboratory data analysis among the groups (*x̅±*s).

Items	Control group (N = 33)	NTH group (N = 30)	TH group (N = 28)	*P* value
IL-6 (pg/mL)	9.20 ± 4.19	18.43 ± 5.37*	30.79 ± 7.63*^,^†	<.05
TF (pg/ mL)	87.62 ± 33.47	309.26 ± 78.46*	421.52 ± 103.73*^,^†	<.05
FXI:C (%)	82.12 ± 34.51	127.64 ± 45.29*	159.48 ± 62.34*^,^†	<.05
D-D(mg/L)	0.35 ± 0.19	1.67 ± 0.32*	2.83 ± 0.67*^,^†	<.05
miR-145	1.06 ± 0.29	0.70 ± 0.18*	0.23 ± 0.09*^,^†	<.05

D-D = D-Dimer, FXI =C = Factor XI concentration, IL-6 = interleukin-6, LAD = left atrial diameter, miR-145 = microRNA-145, NTH = non thrombus, TF = tissue factor, TH = thrombus.

* *P* value < 0.05, Compared with the control group.

† *P* value < 0.05, Compared with NTH group.

### 3.3. Logistic regression

Logistic regression analysis showed that the elevated Factor XI concentration (OR = 3.60, 95% CI: 1.218–10.637, *P* < .05), the elevated TF level (OR = 3.75, 95% CI: 1.251–11.244, *P* < .05), the elevated D-D level (OR = 3.92, 95% CI: 1.281–12.017, *P* < .05), the elevated IL-6 level (OR = 4.29, 95% CI: 1.413–13.068, *P* < .05), and the decreased miR-145 expression (OR = 4.01, 95% CI: 1.284–12.465, *P* < .05) were the independent risk factors for intracardiac thrombosis in the patients with RHD.

### 3.4. Correlation analysis

**Figure 4.**
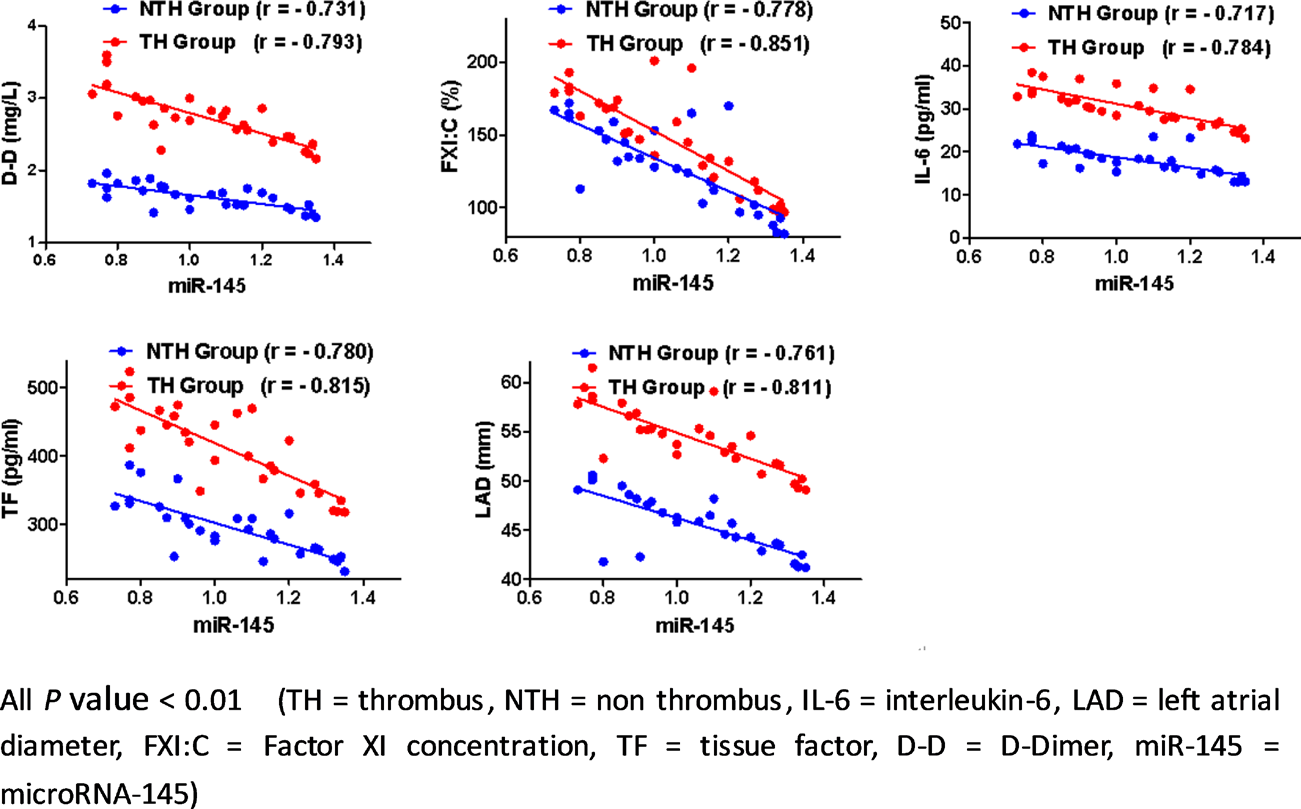
Confirmation of correlation analysis.

In TH group and NTH group, the expression of miR-145 was negatively correlated with D-D level, FXI: C and TF level as well as LAD (all *P* < .01, Fig. 4).

### 3.5. ROC curve analysis

**Figure 5.**
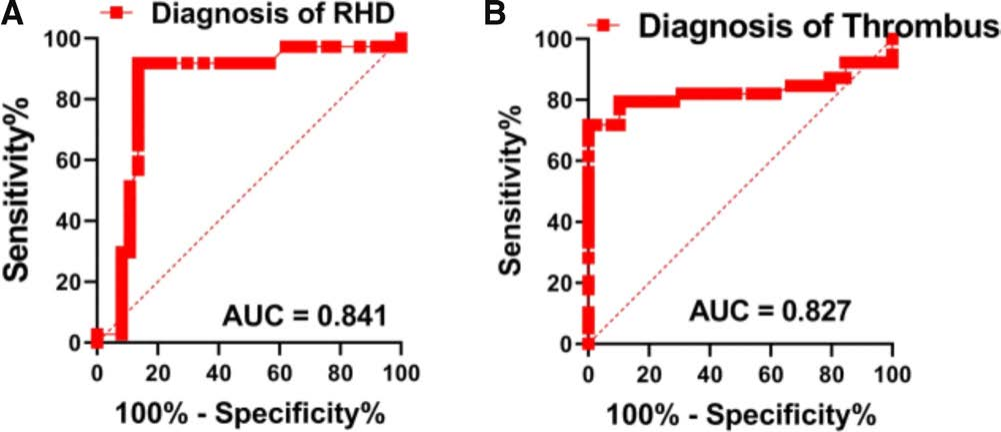
Confirmation of ROC analysis. ROC = receiver operating curve.

The area under curve of miR-145 was (0.841 [95% CI: 0.736–0.947, *P* < .01], Fig. 5) with the data of TH group and NTH group as dependent variables. The sensitivity and specificity of miR-145 in the diagnosis of RHD were 91.89% and 86.49%, respectively.

The area under curve of miR-145 was (0.827 [95% CI: 0.719–0.934, *P* < .01], Fig. 5) with the data of NTH group as dependent variables. The sensitivity and specificity of miR-145 in the diagnosis of thrombosis were 73.79% and 97.44%, respectively.

## 4. Discussion

Studies have found that RHD is caused by recurrent acute rheumatic fever, which may lead to severe heart valve injury, hemodynamic changes, ventricular remodeling and subsequent heart failure, pulmonary hypertension, AF, thromboembolism, infective endocarditis, and may eventually lead to premature death.^[10]^

Rheumatic valvular disease is an autoimmune inflammatory process caused by group A streptococcal infection. A persistent subclinical inflammatory response contributes to the progression of heart valve injury. There is an evidence that the patients with the chronic rheumatic heart valve disease are characterized by an increase in the inflammatory factors such as IL-6.^[11]^ This is consistent with our conclusion. In the early stage of RHD, the cardiac valve lesions caused by inflammatory response mainly include mitral regurgitation and/ or stenosis. Both of these conditions will lead to a significant increase in left atrial volume and pressure load, resulting in left atrial enlargement, which is the main mechanism of left atrial structural remodeling in the early stage of RHD.^[12]^ At the same time, the slowing and stagnation of left atrial blood flow associated with rheumatic mitral stenosis can lead to intracardiac thrombosis.^[13]^ At present, Left atrium (LA) dilation, myocardial remodeling and hemodynamic changes are the important mechanisms of LA thrombosis.^[14]^ In this study, our conclusion also confirmed the fact of LA expansion in RHD patients. We further found that the left atrial diameter and IL-6 level in RHD patients with thrombosis showed a significant increase trend compared with those without thrombosis, suggesting that the changes of left atrial diameter and IL-6 level may be related to the formation of intracardiac thrombosis in RHD.

Elevated plasma D-D levels are considered to be a marker of enhanced coagulation system and active fibrinolysis system.^[15–17]^ One study showed that D-D level was significantly elevated in the patients with rheumatic mitral stenosis.^[18]^ Heppell et al^[19]^ Found that the level of D-D in LA thrombosis patients with rheumatic AF was higher than that in non-thrombosis Group. Normal D-D level has a very high negative predictive value for LA thrombosis.^[20]^ In this study, the level of D-D in the control group, NTH group and TH group showed a gradual increasing trend, indicating that RHD patients had a risk of thrombosis, and the seriously increased level of D-D predicted the occurrence of thrombosis. MicroRNA was reported to be associated with autoimmune diseases. The abnormal expression of microRNA could be related to the pathological process of inflammatory heart valve. For example, inhibition of miR-155-5p could reduce the level of IL-6 in heart valves and serum in RHD patients and reduce valve inflammation and fibrosis induced by RHD.^[21,22]^ Previous studies have demonstrated the antioxidant and anti-inflammatory effects of miR-145,^[23]^ such as the downregulation of miR-145-5p expression in left and right atrial appendages in patients with rheumatic mitral valve disease.^[8]^ A correlation between miR-145 and IL-6 was also reported. The introduction of miR-145-5p can effectively inhibit the production of inflammatory factors in cardiomyocytes induced by hypoxia, including IL-1β, TNF- α, and IL-6.^[24]^ TNF-α treatment can significantly induced human aortic endothelial cells to release inflammatory cytokines, including IL-6 and IL-8, while reducing the expression of miR-145.^[25]^ In this study, we found that the expression of miR-145 decreased in RHD patients, especially in TH group. Interestingly, we found that miR-145 was negatively correlated with LAD, IL-6 level, and D-D level, respectively, suggesting that miR-145 may be involved in the pathogenesis of RHD, and the significant decrease of miR-145 expression can predict intracardiac thrombosis in patients with RHD.

Atrial fibrillation is the most persistent arrhythmia in the general population and is the main problem of RHD.^[26]^ It is a factor in the progression and decompensation of RHD heart failure.^[10,27–29]^ However, the pathogenesis of AF is not completely clear. Current studies have shown that atrial remodeling in patients with RHD may be related to the occurrence and maintenance mechanism of AF. Atrial remodeling includes atrial electrical remodeling (AER) and atrial anatomical remodeling. Changes in the structure, function and characteristics of atrial ion channels are considered to be the key mechanism of AER, in which L-type calcium channels play an important role in regulating atrial rhythm and changes in atrial action potential.^[4]^ Intracellular calcium overload is considered to be a trigger of atrial remodeling. In addition, atrial fibrosis and atrial myocyte apoptosis can lead to atrial enlargement, and hint the occurrence of AER. On the contrary, AER can also maintain the progression of AF.^[30]^

MiRNA is thought to be involved in cardiac electrical remodeling. In the canine AF model induced by injection of hyper propionic acid, the expression of miR-145 in atrial tissue decreased by at least 50%. MiR-145 has been identified as a potential regulator of CACNA1C and CACNB2 genes encoding cardiac L-type calcium channels, and the downregulation of the channel activity is found to be brought about by the increased expression of miR-145,^[31]^ suggesting that miR-145 is related to the occurrence and maintenance of AF.

Studies suggested that AF and rheumatic mitral stenosis, especially when they were combined, increased the risk of LA or ventricular thrombosis and systemic embolism. In particular, intracardiac embolus shedding was a serious complication that could lead to serious cardiovascular adverse events, including ischemic diseases, such as acute myocardial infarction and stroke. During mitral stenosis and/ or AF, the level of D-D increased significantly,^[32,33]^ suggesting that patients with RHD have enhanced coagulation function and active fibrinolytic system. In addition, AF has been proved to be a recognized risk factor for thromboembolic complications caused by left atrial appendage thrombosis, including ischemic stroke.^[34]^

FXI is a coagulation factor involved in the composition of coagulation pathway, which has been proved to be related to the pathogenesis of thrombosis.^[35]^ Elevated plasma FXI levels increased the risk of venous thromboembolism (VTE) and ischemic stroke in some^[36–39]^ studies, but not all^[40]^ studies. In the patients with severe FXI deficiency caused by some mutations in FXI gene, the incidence of VTE and stroke is reduced.^[39,41,42]^ A large number of genetic studies have shown that the genetic variation or deletion of FXI gene is related to the occurrence of VTE^[43–47]^ and ischemic stroke.^[48]^ In nonhuman primate models, FXI seemed to be a more important driver of thrombosis than FXII, and anti FXI antibodies seemed to attenuate platelet and fibrin deposition more than anti FXII antibodies, regardless of whether coagulation stimulation at the site of plaque rupture or left atrial appendage was driven by TF or polyphosphate activated FXII.^[49,50]^ Inhibition of FXI can not only affect the stability of thrombus, but also better down regulate the coagulation activity of patients with AF and further reduce the risk of bleeding.^[51]^ Current studies have shown that miR-145 has been identified to target FXI mRNA and regulate FXI level through its 3 - UTR.^[52]^ Therefore, it is considered that the overexpression of miR-145 has an inhibitory effect on thrombosis. In this study, we determined that plasma FXI: C in patients with RHD complicated with AF was higher than that in the control group, while plasma FXI: C in patients with thrombosis tended to be higher. Our further study showed that miR-145 was associated with FXI: C, and the differential expression of miR-145 suggested the risk of thrombosis in patients with AF. AF complicated with thrombosis is a process with complex influencing factors. Immune cells are thought to induce thrombosis. However, the potential mechanism by which they induce thrombosis is unclear.^[53]^ TF is the main trigger factor of coagulation in vivo. TF binds to the complex activator Factor IX and Factor X of Factor VIIa, resulting in the formation of thrombin.^[54]^ Tumor suppressor is an important cytokine secreted by macrophages, which has been proved to promote the progress of inflammatory response, induce atrial intimal smooth muscle cells in AF patients to secrete TF and inhibit the expression of TF pathway inhibitors, so as to increase the activity of exogenous thrombin and eventually lead to thrombosis.^[53]^ It is reported that endothelial progenitor cells can promote the recanalization of thrombosis. Injecting miR-145 transfected endothelial progenitor cells into the cerebral infarction mouse model can promote the proliferation and migration of mouse endothelial progenitor cells and the recanalization of arterial thrombosis through Jun N-terminal kinase signaling pathway.^[55]^ Further studies showed that the level of miR-145 was negatively correlated with the thrombus load reflecting coagulation activity. It is considered that miRNA targeting prediction tools and in vitro studies have determined that TF is the target gene of miR-145. In thrombotic animal experiments, increasing miR-145 level by simulating in vivo transport could lead to decreased TF level and reduced thrombosis.^[56]^ In this study, we determined that TF level significantly elevated in patients with RHD complicated with AF, especially in the TH group. In addition, we also found that TF level was significantly correlated with the expression of miR-145, suggesting that miR-145 may be involved in the mechanism of thrombosis in RHD patients.

## 5. Conclusion

In this study, we found that the increased levels of LAD, IL-6, D-D, TF as well as FXI: C, and the decreased expression of miR-145 were the risk factors of intracardiac thrombosis for RHD patients. The analysis of ROC curve showed that miR-145 had diagnostic significance for intracardiac thrombosis in patients with RHD, and had high sensitivity and specificity. Therefore, we believe that detecting the expression of miR-145 can predict intracardiac thrombosis in patients with RHD.

## 6. Limitations

Firstly, in this study, the clinical characteristics of patients were analyzed for RHD complicated with AF, and did not analyze for RHD without AF. Second, this study only analyzed the effects of TF and FXI on RHD, and other coagulation factors were not mentioned.

## Acknowledgements

We thank Dr Yanxun Kang from the medical imaging center of First people’s Hospital of Xianyang city for his suggestions and comments on this manuscript.

## Author contributions

**Conceptualization:** Xiong Zhang, Xu-Ai Ren, Xiao-Ming Su.

**Data curation:** Kai Deng.

**Formal analysis:** Xiao-Ming Su.

**Investigation:** Xu-Ai Ren.

**Methodology:** Xiong Zhang.

**Project administration:** Xu-Ai Ren.

**Resources:** Xiao-Ming Su.

**Software:** Kai Deng.

**Writing – original draft:** Kai Deng.
